# Metabolic Syndrome, Sarcopenia and Role of Sex and Age: Cross-Sectional Analysis of Kashiwa Cohort Study

**DOI:** 10.1371/journal.pone.0112718

**Published:** 2014-11-18

**Authors:** Shinya Ishii, Tomoki Tanaka, Masahiro Akishita, Yasuyoshi Ouchi, Tetsuo Tuji, Katsuya Iijima, for the Kashiwa study investigators

**Affiliations:** 1 Department of Geriatric Medicine, Graduate School of Medicine, The University of Tokyo, 7-3-1 Hongo, Bunkyo-ku, Tokyo 113-8655 Japan; 2 Institute of Gerontology, The University of Tokyo, 7-3-1 Hongo, Bunkyo-ku, Tokyo 113-8656, Japan; 3 Federation of National Public Service Personnel Mutual Aid Associations, Toranomon Hospital, 2-2-2, Toranomon, Minato-ku, Tokyo 105-8470, Japan; West Virginia University School of Medicine, United States of America

## Abstract

Recent epidemiological evidence suggests that effects of cardiovascular risk factors may vary depending on sex and age. In this study, we assessed the associations of metabolic syndrome (MetS) with sarcopenia and its components in older adults, and examined whether the associations vary by sex and age. We also tested if any one of the MetS components could explain the associations. We conducted a cross-sectional analysis of the baseline data from the cohort study conducted in Kashiwa city, Chiba, Japan in 2012 which included 1971 functionally-independent, community-dwelling Japanese adults aged 65 years or older (977 men, 994 women). Sarcopenia was defined based on appendicular skeletal muscle mass, grip strength and usual gait speed. MetS was defined based on the National Cholesterol Education Program’s Adult Treatment Panel-III criteria. The prevalence of sarcopenia was 14.2% in men and 22.1% in women, while the prevalence of MetS was 43.6% in men and 28.9% in women. After adjustment for potential confounders, MetS was positively associated with sarcopenia in men aged 65 to 74 years (odds ratio 5.5; 95% confidence interval 1.9–15.9) but not in older men or women. Among the sarcopenia components, MetS was associated with lower muscle mass and grip strength, particularly in men aged 65 to 74 years. The associations of MetS with sarcopenia and its components were mainly driven by abdominal obesity regardless of sex or age. In conclusion, MetS is positively associated with sarcopenia in older men. The association is modified by sex and age, but abdominal obesity is the main contributor to the association across sex and age.

## Introduction

Metabolic syndrome (MetS) is a constellation of cardiovascular risk factors which include abdominal obesity, dyslipidemia, hypertension and elevated glucose [1]. Insulin resistance and chronic inflammation are considered central mechanisms responsible for MetS [2] and inextricably correlate with each other to exert detrimental metabolic effects and lead to cardiovascular morbidity and mortality [3]–[5]. Accumulating epidemiological evidence suggests that both insulin resistance and chronic inflammation cause adverse effects on skeletal muscle. Diabetes, or even insulin resistance without diabetes, is associated with greater declines in skeletal muscle mass and strength [6], [7]. A link between inflammation and muscle weakness has been reported in several studies [8], [9]. Therefore, we postulate that MetS can accelerate age-related loss of muscle mass and strength, leading to the development of sarcopenia, a syndrome characterized by loss of skeletal muscle mass and function with a risk of physical disability [10]. Indeed, recent studies showed that MetS is associated with physical capacity impairment and increased risk of developing physical and functional disabilities [11]–[13].

Several recent studies have suggested that the effects of MetS may vary depending on age and sex. Cardiovascular risk factors, whose adverse effects have been established in younger people, may have different impacts in the elderly or frail population. Obesity did not seem to be a risk factor for increased mortality in elderly hospitalized patients with or without diabetes [14], [15]. Elevated blood pressure was associated with lower mortality risk in physically frail elderly adults who could not walk 20 feet [16]. MetS was associated with lower probability of prevalent and incident functional disability in older adults [17]. The association between MetS and cardiovascular events was observed only in patients younger than 75, but not in patients aged 75 or over [18]. With regard to sex-related differences in the effects of MetS, MetS was associated with lower muscle strength in elderly men but not in elderly women [19]. However, data on sex- or age-related differences in the effect of MetS on sarcopenia are still scarce.

In the present study, we assessed the associations of MetS with sarcopenia and its components in functionally-independent community-dwelling Japanese older adults, and examined whether the associations were modified by sex or age. We hypothesized that MetS is positively associated with sarcopenia and its components, and that the associations are more pronounced in relatively young men. We also examined whether any of the individual MetS components could explain the associations and if the same MetS components contributed to the associations across sex and age.

## Methods

### Subjects

The Kashiwa study is a prospective cohort study designed to characterize the biological, psychosocial and functional changes associated with aging in a community-based cohort of 2044 older adults (1013 men, 1031 women). Those aged 75 and older accounted for 36.3% of men and 35.0% of women. The sampling and data collection process has been described in detail elsewhere [20]. Briefly, the inclusion criteria were age equal to or older than 65 years and functional independence (i.e., not requiring nursing care provided by long-term care insurance). The subjects were randomly selected from the resident register of Kashiwa city, Chiba, Japan, enrolled in 2012, and followed annually. The current study is a cross-sectional analysis of the Kashiwa study baseline data. Seventy three subjects who did not undergo bioimpedance analysis (BIA), usual gait speed or hand grip strength measurements were excluded, leaving an analytic sample of 1971 older adults (977 men, 994 women). Those excluded from the analysis were older compared to those included in the analysis (mean age 75.9 years vs. 72.9 years, p = 0.001), but did not significantly differ with respect to other characteristics including sex, height, weight, and prevalence of MetS.

The study was approved by the ethics committee of the Graduate School of Medicine, The University of Tokyo. All subjects provided written informed consent.

### Definition of Sarcopenia

We followed the recommendations of the European Working Group on Sarcopenia in Older People (EWGSOP) for the diagnostic definition of sarcopenia [10]. The proposed diagnostic criteria required the presence of low muscle mass plus the presence of either low muscle strength or low physical performance. Muscle mass was measured by BIA using an Inbody 430 machine (Biospace, Seoul, Korea). Appendicular skeletal muscle mass (ASM) was derived as the sum of the muscle mass of the four limbs [10]. ASM was then normalized by height in meters squared to yield skeletal muscle mass index (SMI) (kg/m^2^). SMI values lower than two standard deviations below the mean values of young male and female reference groups were classified as low muscle mass (SMI <7.0 kg/m^2^ in men, <5.8 kg/m^2^ in women) [21]. Muscle strength was assessed by hand grip strength, which was measured using a digital grip strength dynamometer (Takei Scientific Instruments, Niigata, Japan). Hand grip strength values in the lowest quintile were classified as low muscle strength in this study (cutoff values: 30 kg for men, 20 kg for women). Physical performance was assessed by usual gait speed. Subjects were instructed to walk over an 11-meter straight course at their usual speed. Usual gait speed was derived from 5 meters divided by the time in seconds spent in the middle 5 meters (from the 3-meter line to the 8-meter line) [22]. Usual gait speed values in the lowest quintile were classified as low physical performance in the current study (cutoff values: 1.26 m/s for each sex).

### Definition of metabolic syndrome

MetS was defined based on the National Cholesterol Education Program Adult Treatment Panel III (NCEP-ATP III) criteria [1]. The presence of any three of the following five abnormalities constitutes a diagnosis of MetS: (i) abdominal obesity; (ii) elevated triglycerides (TG) with fasting plasma triglycerides ≥150 mg/dL; (iii) low high density lipoprotein cholesterol (HDL-C) with fasting HDL-C <40 mg/dL in men and <50 mg/dL in women; (iv) elevated blood pressure with systolic blood pressure ≥130 mmHg and/or diastolic blood pressure ≥85 mmHg; (v) elevated fasting plasma glucose with fasting plasma glucose ≥100 mg/dL. Abdominal obesity was defined by waist circumference using the thresholds recommended by the Japanese Obesity Society (≥85 cm in men and ≥90 cm in women) [1].

Waist circumference was measured at the umbilical level using a measuring tape with the subject in an upright position. Blood pressure was measured using a standard technique with an HEM-7080IT automated measuring device (Omron Co., Tokyo, Japan). Blood samples were obtained after an overnight fast. Total cholesterol, HDL-C and TG were analyzed by enzymatic methods using a JCA-BM8060 automated analyzer (Japan Electron Optics Laboratory Ltd., Tokyo, Japan). Fasting plasma glucose level was measured using a JCA-BM9030 automated analyzer (Japan Electron Optics Laboratory Ltd.).

### Other measurements

Demographic information, medical history of doctor-diagnosed chronic conditions, use of medication, and food intake were obtained using a standardized self-reported questionnaire. Physical activity was assessed using the Global Physical Activity Questionnaire, and metabolic equivalents (METs)-minute per week was computed [23]. Height and weight were measured with the subject wearing light clothing and no shoes using a fixed stadiometer and a digital scale, and used to compute body mass index (BMI).

### Statistical Analysis

Differences in subject characteristics between those with and without sarcopenia were examined using Student’s t-test or Wilcoxon rank-sum test (for continuous variables) and chi-square test (for categorical variables).

First, we employed logistic regression analysis to evaluate the association of MetS with sarcopenia. Our preliminary analysis suggested that the association of metabolic syndrome with sarcopenia was modified by sex (p<0.01), and therefore the following analyses were stratified by sex.

The model was initially adjusted for age only (model 1). We added height and weight to remove the confounding effect of body size (model 2). We then further adjusted for life-style risk factors for both sarcopenia and MetS, including physical activity and food intake (model 3). In the fully-adjusted model, the interaction between MetS and age was examined to test the hypothesis that the effect of MetS on sarcopenia varies by age.

To test if any MetS component could explain the MetS-sarcopenia association, we initially fitted a fully-adjusted logistic regression model to examine the association between each component of MetS and sarcopenia, followed by other logistic regression models between MetS and sarcopenia adjusted for MetS components.

Second, to examine the association of MetS with each component of sarcopenia (i.e., muscle mass, grip strength and usual gait speed), we employed multiple linear regression models. If the association between MetS and any one of the sarcopenia components was statistically significant, another multiple linear regression model with MetS components as independent variables instead of MetS was conducted to evaluate the association between MetS components and the sarcopenia component. Finally, each component of MetS was introduced as a covariate to the multiple linear regression model between MetS and the sarcopenia component to test if the MetS component could explain the association between MetS and the sarcopenia component. Considering that the number of combinations between MetS components and sarcopenia components is quite high, the analyses between MetS components and sarcopenia components were considered supplemental and carried out only when the association between MetS and any of the sarcopenia components was statistically significant, in order to decrease the possibility of finding associations that were significant just by chance alone.

There were no missing values of any variable in the entire analytic sample.

All analyses were conducted using SAS version 9.3 (SAS Institute Inc., Cary, NC) and R statistical software version 2.15.2 (R Foundation, Vienna, Austria). Two-sided p<0.05 was considered statistically significant.

## Results

### Subject characteristics

The prevalence of sarcopenia was 14.2% in men and 22.1% in women, and 43.6% of men and 28.9% of women were classified as having MetS. The characteristics of the study subjects by the sarcopenia status in each sex are shown in Table 1. Those with sarcopenia were older and had smaller body size compared with those without sarcopenia in each sex. Those with sarcopenia were physically less active and had smaller food intake in each sex. The prevalence of MetS was higher in those without sarcopenia, but the difference was significant only in men (p = 0.048 in men, 0.052 in women). Among the five MetS components, abdominal obesity was significantly more prevalent in those without sarcopenia in each sex.

**Table 1:** Characteristics of all subjects and according to sarcopenia status in men and women.

	All	Sarcopenia	No sarcopenia	p
Men	977	139 (14.2%)	838 (85.8%)	
Age (years)	73.1±5.5	78.4±5.5	72.2±5.0	<0.001
Height (cm)	164.2±5.8	160.0±5.6	164.9±5.5	<0.001
Weight (kg)	62.8±8.6	54.1±7.2	64.3±8.0	<0.001
BMI (kg/m^2^)	23.3±2.8	21.1±2.5	23.6±2.6	<0.001
SMI (kg/m^2^)	7.28±0.68	6.34±0.48	7.44±0.58	<0.001
Hand grip strength (kg)	34.8±6.0	27.5±4.3	36.0±5.3	<0.001
Usual gait speed (m/s)	1.47±0.26	1.28±0.24	1.51±0.24	<0.001
MetS	43.6%	36.0%	44.9%	0.048
MetS components				
Abdominal obesity	55.5%	36.0%	58.7%	<0.001
High TG	22.7%	21.6%	22.9%	0.73
Low HDL-C	21.4%	20.9%	21.5%	0.87
High BP	90.4%	88.5%	90.7%	0.41
High FPG	51.0%	53.2%	50.6%	0.56
Food intake				
Very large	2.9%	1.4%	3.1%	<0.001
Large	15.3%	5.8%	16.8%	
Normal	65.4%	58.3%	66.6%	
Small	14.4%	30.2%	11.8%	
Very small	2.1%	4.3%	1.7%	
Physical activity (Mets)	3962.9±3981.0	3191.7±3612.2	4090.8±4026.7	0.01
Medical history				
Hypertension	47.2%	51.1%	46.5%	0.32
Diabetes	15.4%	18.0%	14.9%	0.36
Dyslipidemia	29.8%	31.7%	29.5%	0.60
Stroke	7.2%	12.2%	6.4%	0.01
CAD	8.0%	11.5%	7.4%	0.10
Cancer	19.0%	26.6%	17.8%	0.01
Medication use				
Statin	17.6%	18.7%	17.4%	0.71
**Women**	**994**	**220 (22.1%)**	**774 (77.9%)**	
Age (years)	72.8±5.4	76.2±5.8	71.8±4.9	<0.001
Height (cm)	151.4±5.5	148.2±5.6	152.3±5.1	<0.001
Weight (kg)	51.5±7.7	46.4±5.7	52.9±7.6	<0.001
BMI (kg/m^2^)	22.5±3.2	21.1±2.6	22.8±3.2	<0.001
SMI (kg/m^2^)	5.84±0.65	5.25±0.41	6.02±0.60	<0.001
Hand grip strength (kg)	22.4±3.9	18.4±3.2	23.6±3.3	<0.001
Usual gait speed (kg)	1.46±0.26	1.26±0.26	1.51±0.23	<0.001
MetS	28.9%	23.6%	30.4%	0.052
MetS components				
Abdominal obesity	24.0%	14.6%	26.7%	<0.001
High TG	17.9%	16.4%	18.4%	0.50
Low HDL-C	36.6%	33.2%	37.6%	0.23
High BP	84.2%	87.3%	83.3%	0.16
High FPG	33.7%	34.1%	33.6%	0.89
Food intake				
Very large	2.0%	1.4%	2.2%	<0.001
Large	13.1%	9.6%	14.1%	
Normal	72.4%	64.1%	74.8%	
Small	11.2%	20.9%	8.4%	
Very small	1.3%	4.1%	0.5%	
Physical activity (Mets)	3722.7±3429.5	2748.0±2825.0	4000.0±3535.6	<0.001
Medical history				
Hypertension	39.8%	45.9%	38.1%	0.04
Diabetes	8.8%	8.2%	8.9%	0.73
Dyslipidemia	46.9%	45.5%	47.3%	0.63
Stroke	4.7%	5.9%	4.4%	0.35
CAD	4.9%	5.5%	4.8%	0.68
Cancer	11.2%	11.8%	11.0%	0.73
Medication use				
Statin	30.3%	29.1%	30.6%	0.66

Mean and standard deviation are shown for continuous variables, and proportions as percent for categorical variables. Percentages may not add up to 100 because of rounding.

Abbreviations: BMI, body mass index; SMI, skeletal muscle mass index; MetS, metabolic syndrome; TG, triglycerides; CAD, coronary artery disease; HDL-C, high density lipoprotein cholesterol; BP, blood pressure; FPG, fasting plasma glucose.

### Association between MetS and sarcopenia

In multiple logistic regression adjusted for age, MetS was significantly associated with *decreased* risk of sarcopenia in each sex (Table 2, Model 1). However, after additional adjustment for body size (i.e., height and weight), MetS was significantly associated with *increased* risk of sarcopenia in men, while the association between MetS and sarcopenia became non-significant in women (Table 2, Model 2). Further adjustment for life-style risk factors had little effect on the association (Table 2, Model 3). Exclusion of subjects who did not meet the criteria for MetS but had one or two MetS components (i.e., comparing those with MetS and those with *no* MetS component) yielded stronger MetS-sarcopenia association in men (OR 8.25, 95% CI 2.17–31.37, p = 0.002), but the association remained non-significant in women (OR 1.10, 95% CI 0.48–2.94, p = 0.83). In the fully adjusted model, the interaction between MetS and age was statistically significant in men (p = 0.02), suggesting that the effect of MetS on sarcopenia may vary by age. We then divided the subjects into two groups according to age: “young old” (65–74 years) and “old old” (≥75 years). The characteristics of the subjects by the sarcopenia status in each subgroup (young-old and old-old) are shown in Table S1. In the age-stratified analysis, MetS was significantly associated with sarcopenia in “young old” men only (Table 2, Model 3b).

**Table 2:** Adjusted associations of metabolic syndrome with sarcopenia in men and women.

	Men		Women	
	OR (95% CI)	p	OR (95% CI)	p
**Model 1**	0.58 (0.38, 0.87)	0.008	0.55 (0.38, 0.79)	0.001
**Model 2**	2.05 (1.21, 3.47)	0.007	1.06 (0.69, 1.65)	0.79
**Model 3**	2.08 (1.22, 3.54)	0.007	1.03 (0.66, 1.61)	0.89
**Model 3a**	1.49 (0.80, 2.76)	0.21	1.02 (0.57, 1.85)	0.94
**Model 3b**	4.99 (1.73, 14.40)	0.003	1.03 (0.52, 2.04)	0.93

Abbreviations: OR, odds ratio; CI, confidence interval.

Model 1: adjusted for age.

Model 2: adjusted for age, height and weight.

Model 3: adjusted for age, height, weight, physical activity and food intake.

Model 3a: Adjusted for the same covariates as in Model 3, restricted to those aged 75 or over.

Model 3b: Adjusted for the same covariates as in Model 3, restricted to those aged 65 to 74.

### Associations of MetS components with sarcopenia

**Figure 1:**
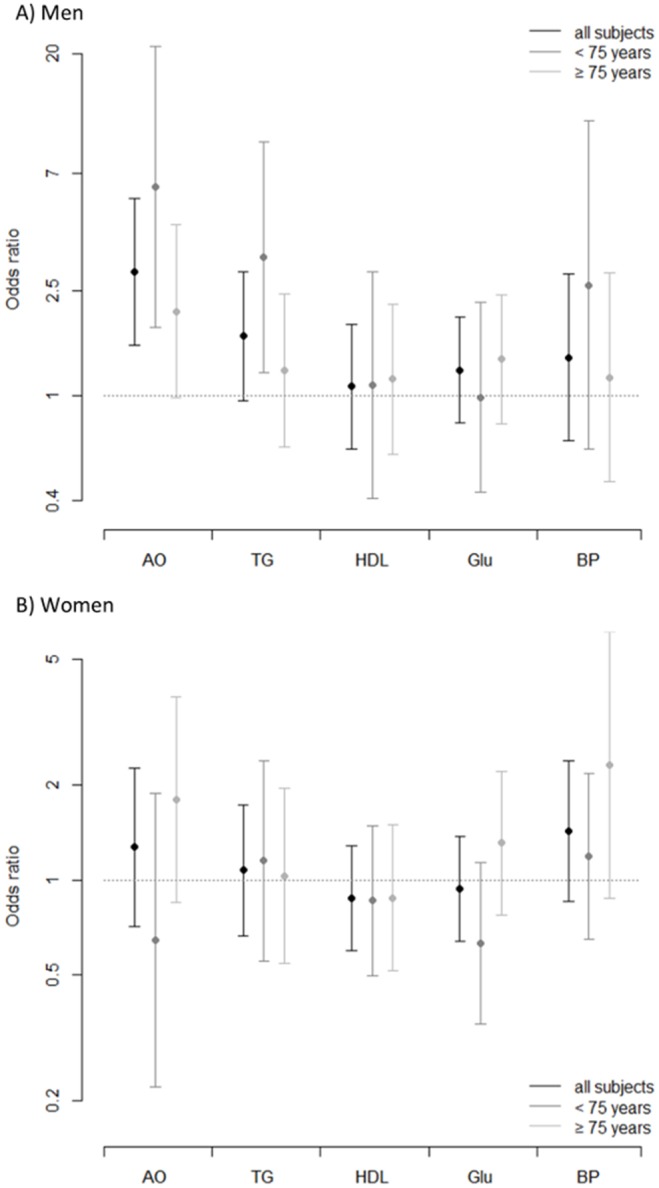
Fully adjusted odds ratio and 95% confidence interval of sarcopenia by individual metabolic syndrome components in all subjects and according to age group. Black bars: all subjects, dark-gray bars: subjects aged 65 to 74 years, light-gray bars: subjects aged 75 years or over. All models are adjusted for age, height, weight, physical activity and food intake. AO, abdominal obesity; TG, elevated triglycerides; HDL, low high density lipoprotein; Glu, elevated fasting plasma glucose; BP, high blood pressure. A) Men. B) Women.

Multiple logistic regression models demonstrated that, of the five MetS components, only abdominal obesity was significantly associated with increased risk of sarcopenia in men (odds ratio [OR] 2.98, 95% confidence interval 1.55–5.63, p≤0.001) while none of the MetS components was significantly associated with sarcopenia in women (Figure 1). Abdominal obesity was significantly and independently associated with sarcopenia in men in the model including all five MetS components simultaneously (OR 2.89, 95% CI 1.51–5.53, p = 0.001). When abdominal obesity was added as a covariate to the logistic regression model between MetS and sarcopenia, the MetS-sarcopenia association became statistically non-significant (p = 0.12), suggesting that the MetS-sarcopenia association was mainly mediated by abdominal obesity. In the age-stratified analysis, abdominal obesity and elevated TG were significantly associated with sarcopenia (OR 6.22, 95% CI 1.82–21.22, p = 0.004 and OR 3.37, 95% CI 1.23–9.28, p = 0.02, respectively) in young-old men, but no significant associations were observed between MetS components and sarcopenia in old-old men or women. Abdominal obesity and elevated TG remained significantly associated with sarcopenia in young-old men in the model including all five MetS components simultaneously (OR 6.32, 95% CI 1.81–22.06, p = 0.004 and OR 3.30, 95% CI 1.19–9.13, p = 0.02, respectively). Addition of abdominal obesity and elevated TG to the model between MetS and sarcopenia in young-old men made the MetS-sarcopenia association statistically non-significant (p = 0.13).

### Associations of MetS with sarcopenia components

In fully-adjusted multiple linear regression models, MetS was associated with lower grip strength in each sex and lower muscle mass in men (Table 3). When analysis was stratified by age, the inverse associations of MetS with muscle mass and grip strength in men remained significant except for the association between MetS and muscle strength in the old-old group, which became statistically non-significant (Table 3). In women, the inverse association between MetS and grip strength was observed in the old-old group only. The association between MetS and muscle mass became significant in old-old women in the age-stratified analysis.

**Table 3:** Adjusted associations of metabolic syndrome with individual sarcopenia components in all subjects and according to age groups in men and women* †.

	Men		Women	
	beta (95% CI)	p	beta (95% CI)	p
**Skeletal muscle mass index**				
All	−0.14 (−0.20, −0.09)	<0.001	−0.05 (−0.10, 0.007)	0.09
Old-old	−0.13 (−0.24, −0.03)	0.009	−0.10 (−0.19, −0.005)	0.04
Young-old	−0.15 (−0.22, −0.08)	<0.001	−0.02 (−0.09, 0.05)	0.57
**Grip strength**				
All	−0.98 (−1.68, −0.28)	0.006	−0.61 (−1.11, −0.10)	0.02
Old-old	−0.65 (−1.76, 0.45)	0.25	−0.84 (−1.64, −0.05)	0.04
Young-old	−1.26 (−2.17, −0.34)	0.007	−0.38 (−1.04, 0.27)	0.25
**Usual gait speed**				
All	−0.02 (−0.06, 0.01)	0.22	−0.01 (−0.05, 0.02)	0.55
Old-old	−0.006 (−0.06, 0.05)	0.83	−0.03 (−0.08, 0.03)	0.36
Young-old	−0.03 (−0.07, 0.009)	0.13	0.004 (−0.04, 0.05)	0.86

Abbreviations; CI, confidence interval.

*All the models were adjusted for age, height, weight, physical activity and food intake.

† The young-old group refers to those aged 65 to 74 and the old-old group to those aged 75 or older.

In the subsequent supplementary analysis, abdominal obesity was significantly associated with lower grip strength in each sex and with lower muscle mass in men (Table S2). In addition, low HDL-C was associated with lower grip strength, and high TG was associated with lower muscle mass in men. These associations observed in men were significant in the young-old group only in the age-stratified analysis. For women, the only significant association observed was between high TG and lower muscle mass in the old-old group.

The association between MetS and grip strength became statistically non-significant after introduction of abdominal obesity into the model in each age group and sex. The introduction of abdominal obesity attenuated the association between MetS and muscle mass (i.e., decreased the magnitude of the regression coefficient) in each age group and sex by more than 10%, more markedly than did any other MetS component, consistent with abdominal obesity dominating the association of MetS with sarcopenia components (data not shown).

## Discussion

In this cross-sectional analysis of 1971 functionally-independent, community-dwelling adults older than 65, MetS was associated with *increased* risk of sarcopenia, particularly in “young-old” men (aged 65 to 74), after adjustment for potential confounders including body size. Without adjustment for body size, MetS was associated with *decreased* risk of sarcopenia, suggesting that body size can confound the association between MetS and sarcopenia and should be taken into account when considering the impact of cardiovascular risk factors on muscle.

We demonstrated that MetS was associated with lower muscle mass and lower muscle strength, but the effects varied by sex and age. The adverse effects of MetS on muscle mass and strength were mainly observed in the young-old group for men. In stark contrast, women were mostly insusceptible to adverse effects of MetS on muscle, except for the marginally statistically significant associations of MetS with muscle mass and strength in the old-old group (age 75 or older). The mechanisms underlying the age- and sex-related differences in the associations between MetS and muscle mass/strength need to be explored in future research, but possible explanations may include the effects of sex hormones on skeletal muscle. MetS is associated with lower testosterone level [24]. Considering that testosterone is positively related to muscle strength [25], it is conceivable that one of the pathways through which MetS exerts its adverse effects on muscle is via testosterone. Since testosterone decreases with age [26] and is lower in women than in men, younger men, with relatively high levels of testosterone, may be especially vulnerable. Another possible explanation is cytokines secreted by adipose tissue, so-called adipokines. Adipose tissue produces and releases adipokines such as adiponectin and leptin as well as pro-inflammatory cytokines such as IL-6 [27]. Skeletal muscle is an important target tissue for these molecules, and circulating levels of such molecules are influenced by the amount of adipose tissue as well as age and sex [28], [29].

Several studies have reported an inverse association between MetS and muscle strength in younger men and women [30], [31]. One small cross-sectional study of older adults revealed an inverse association between MetS and muscle strength in men, but not in women [19]. This study also demonstrated that the association between MetS and muscle strength was more pronounced in men aged 65–74 compared to men aged 75 or older, consistent with our findings. Low muscle mass, with or without the presence of obesity, is associated with MetS in younger men and women [32]–[34]. Several studies in older adults showed an inverse association between MetS and muscle mass [35], [36], but these studies did not assess men and women separately.

We also demonstrated that the observed associations of MetS with the summary definition of sarcopenia or its individual components were mainly driven by abdominal obesity regardless of sex and age. Neither high BP nor elevated FPG showed a statistically significant association with sarcopenia or its components. Only a few studies have assessed which MetS components are main contributors to the association between MetS and the summary definition of sarcopenia or its components. An inverse association between MetS and physical performance was found in the cross-sectional analysis of a large-scale cohort study of older men, with obesity having the highest regression coefficient on physical performance among five MetS components [37]. Likewise, another large-scale cohort study of older adults found an association between MetS and poor physical performance, with abdominal obesity explaining the largest fraction of the variation in physical performance [38]. Our findings confirmed these previous studies and additionally demonstrated that abdominal obesity may be the main contributing factor for the associations of MetS with sarcopenia and its individual components regardless of sex and age, suggesting that there is a common mechanism underlying the adverse effects of MetS on muscle, for which abdominal obesity may partly be a marker, and that additional factors are at play causing sex- and age-related differences. Visceral fat accumulation, or abdominal obesity, is hypothesized to play an essential role in the development of MetS, given its propensity to cause insulin resistance, chronic inflammation and lower adiponectin levels [39]–[42]. All these factors may also be involved in the pathophysiological process of development of sarcopenia [6]–[9], [28], and we postulate that abdominal obesity may represent a clinical phenotype that is associated with increased risk of developing both MetS and sarcopenia. This study had several limitations. First, it could not be free of unmeasured or uncontrolled confounders due to its observational nature. In addition, since this study was cross-sectional, we could not infer a causal relationship between MetS and sarcopenia. Low muscle mass is associated with physical inactivity [10] and insulin resistance [43], and therefore could lead to the development of MetS. We speculate that, in reality, sarcopenia and MetS are deeply intertwined and cause adverse effects on each other, leading to frequent co-existence of these two syndromes. Second, medical history, use of medication and food intake were self-reported. Even though we used a standardized questionnaire, reporting bias was possible. Third, we did not collect information on or adjust for food composition such as total calories, which may confound the sarcopenia-MetS association. Finally, since the subjects were exclusively functionally-independent Japanese older adults, our findings may not be able to be generalized to older adults from other racial/ethnic groups.

In conclusion, this study comprehensively examined the associations of MetS with sarcopenia and its individual components in older adults, with particular attention to the modifying effects of sex and age. We demonstrated associations of MetS with sarcopenia, particularly muscle mass and strength. The associations were modified by sex and age, but were mainly driven by abdominal obesity regardless of sex and age. This study adds to the growing knowledge on the adverse effects of MetS on muscle. Further research is needed to elucidate the underlying mechanisms of the sex- and age-related differences in the association between MetS and sarcopenia.

## Supporting Information

Table S1 **Characteristics of subjects according to sarcopenia status and age in men and women.**
(DOCX)

Table S2 **Adjusted associations of metabolic syndrome components with individual sarcopenia components.**
(DOCX)
