# Supplementary material for: Metabolic Syndrome, Sarcopenia and Role of Sex and Age: Cross-Sectional Analysis of Kashiwa Cohort Study
Source: PLoS One. 2014 Nov 18;9(11):e112718. doi: 10.1371/journal.pone.0112718 (PMC4236117; doi:10.1371/journal.pone.0112718)
Supplement: Table S1 — Characteristics of subjects according to sarcopenia status and age in men and women. (DOCX) [file pone.0112718.s001.docx]

Table S1. Characteristics of subjects according to sarcopenia status and age in men and women

|  | Young-old (age 65-74 years) | | | Old-old (age ≥ 75 years) | | |
| --- | --- | --- | --- | --- | --- | --- |
|  | Sarcopenia | No sarcopenia | p | Sarcopenia | No sarcopenia | p |
| Men | 33 (5.3%) | 589 (94.7%) |  | 106 (29.9%) | 249 (70.1%) |  |
| Age (years) | 71.2 ± 2.5 | 69.5 ± 2.6 | <.001 | 80.6 ± 4.2 | 78.5 ± 3.5 | <.001 |
| Height (cm) | 160.4 ± 6.4 | 165.4 ± 5.5 | <.001 | 159.9 ± 5.3 | 163.9 ± 5.5 | <.001 |
| Weight (kg) | 53.9 ± 8.0 | 64.7 ± 7.9 | <.001 | 54.2 ± 7.0 | 63.3 ± 8.2 | <.001 |
| BMI (kg/m^2^) | 20.9 ± 2.6 | 23.6 ± 2.5 | <.001 | 21.2 ± 2.5 | 23.6 ± 2.9 | <.001 |
| SMI (kg/m^2^) | 6.39 ± 0.49 | 7.59 ± 0.56 | <.001 | 6.33 ± 0.48 | 7.32 ± 0.59 | <.001 |
| Hand grip strength (kg) | 28.1 ± 4.1 | 37.1 ± 5.2 | <.001 | 27.3 ± 4.4 | 33.6 ± 4.9 | <.001 |
| Usual gait speed (m/s) | 1.32 ± 0.17 | 1.54 ± 0.23 | <.001 | 1.26 ± 0.26 | 1.44 ± 0.26 | <.001 |
| MetS | 39.4% | 43.0% | 0.69 | 34.9% | 49.4% | 0.01 |
| MetS components |  |  |  |  |  |  |
| Abdominal obesity | 36.4% | 56.9% | 0.02 | 35.9% | 63.1% | <.001 |
| High TG | 24.2% | 22.8% | 0.84 | 20.8% | 23.3% | 0.60 |
| Low HDL-C | 18.2% | 20.0% | 0.80 | 21.7% | 24.9% | 0.52 |
| High BP | 90.9% | 90.0% | 0.86 | 87.7% | 92.4% | 0.16 |
| High FPG | 36.4% | 49.2% | 0.15 | 58.5% | 53.8% | 0.42 |
| Food intake |  |  |  |  |  |  |
| Very large | 0% | 3.4% | <.001 | 1.9% | 2.4% | 0.007 |
| Large | 3.0% | 18.3% |  | 6.6% | 13.3% |  |
| Normal | 57.6% | 66.0% |  | 58.5% | 67.9% |  |
| Small | 39.4% | 10.7% |  | 27.4% | 14.5% |  |
| Very small | 0% | 1.5% |  | 5.7% | 2.0% |  |
| Physical activity (Mets) | 3394.6 ± 3353.9 | 4057.6 ± 4163.3 | 0.37 | 3128.5 ± 3701.8 | 4169.4 ± 3690.2 | 0.02 |
| Medical history |  |  |  |  |  |  |
| Hypertension | 48.5% | 44.1% | 0.63 | 51.9% | 52.2% | 0.96 |
| Diabetes | 9.1% | 14.5% | 0.39 | 20.8% | 16.1% | 0.29 |
| Dyslipidemia | 33.3% | 28.7% | 0.57 | 31.1% | 31.3% | 0.97 |
| Stroke | 6.1% | 5.1% | 0.81 | 14.2% | 9.2% | 0.17 |
| CAD | 9.1% | 7.3% | 0.70 | 12.3% | 7.6% | 0.16 |
| Cancer | 18.2% | 15.3% | 0.65 | 29.3% | 23.7% | 0.27 |
| Medication use |  |  |  |  |  |  |
| Statin | 18.2% | 15.8% | 0.71 | 18.9% | 21.3% | 0.61 |
|  | Young-old (age 65-74 years) | | | Old-old (age ≥ 75 years) | | |
|  | Sarcopenia | No sarcopenia | p | Sarcopenia | No sarcopenia | p |
| Women | 91 (14.1%) | 555 (85.9%) |  | 129 (37.1%) | 219 (62.9%) |  |
| Age (years) | 70.6 ± 2.4 | 69.3 ± 2.7 | <.001 | 80.2 ± 3.9 | 78.2 ± 3.1 | <.001 |
| Height (cm) | 149.4 ± 5.5 | 152.9 ± 5.1 | <.001 | 147.3 ± 5.5 | 151.0 ± 5.1 | <.001 |
| Weight (kg) | 45.9 ± 5.8 | 53.0 ± 7.7 | <.001 | 46.7 ± 5.6 | 52.8 ± 7.2 | <.001 |
| BMI (kg/m^2^) | 20.6 ± 2.6 | 22.7 ± 3.2 | <.001 | 21.6 ± 2.6 | 23.2 ± 3.3 | <.001 |
| SMI (kg/m^2^) | 5.28 ± 0.37 | 6.04 ± 0.61 | <.001 | 5.23 ± 0.44 | 5.96 ± 0.58 | <.001 |
| Hand grip strength (kg) | 18.5 ± 3.1 | 24.0 ± 3.3 | <.001 | 18.3 ± 3.3 | 22.3 ± 3.1 | <.001 |
| Usual gait speed (kg) | 1.37 ± 0.24 | 1.54 ± 0.22 | <.001 | 1.17 ± 0.23 | 1.44 ± 0.24 | <.001 |
| MetS | 14.3% | 25.8% | 0.02 | 30.2% | 42.0% | 0.03 |
| MetS components |  |  |  |  |  |  |
| Abdominal obesity | 5.5% | 23.6% | <.001 | 20.9% | 34.7% | 0.007 |
| High TG | 13.2% | 17.1% | 0.35 | 18.6% | 21.5% | 0.52 |
| Low HDL-C | 27.5% | 33.9% | 0.23 | 37.2% | 47.0% | 0.07 |
| High BP | 78.0% | 80.5% | 0.58 | 93.8% | 90.4% | 0.27 |
| High FPG | 22.0% | 29.9% | 0.12 | 42.6% | 42.9% | 0.96 |
| Food intake |  |  |  |  |  |  |
| Very large | 1.1% | 2.5% | 0.006 | 1.6% | 1.4% | <.001 |
| Large | 12.1% | 13.9% |  | 7.8% | 14.6% |  |
| Normal | 67.0% | 75.7% |  | 62.0% | 72.6% |  |
| Small | 17.6% | 7.6% |  | 23.3% | 10.5% |  |
| Very small | 2.2% | 0.4% |  | 5.4% | 0.9% |  |
| Physical activity (Mets) | 3025.3 ± 2865.6 | 3988.9 ± 3467.9 | 0.01 | 2552.4 ± 2790.6 | 4027.3 ± 3709.7 | <.001 |
| Medical history |  |  |  |  |  |  |
| Hypertension | 30.8% | 34.1% | 0.54 | 56.6% | 48.4% | 0.14 |
| Diabetes | 6.6% | 8.8% | 0.48 | 9.3% | 9.1% | 0.96 |
| Dyslipidemia | 39.6% | 45.4% | 0.30 | 49.6% | 52.1% | 0.66 |
| Stroke | 4.4% | 3.6% | 0.71 | 7.0% | 6.4% | 0.83 |
| CAD | 3.3% | 3.6% | 0.88 | 7.0% | 7.8% | 0.79 |
| Cancer | 5.5% | 9.7% | 0.19 | 16.3% | 14.2% | 0.59 |
| Medication use |  |  |  |  |  |  |
| Statin | 25.3% | 27.4% | 0.67 | 31.8% | 38.8% | 0.19 |

Mean and standard deviation are shown for continuous variables, and proportions as percent for categorical variables. Percentages may not add up to 100 because of rounding.

Abbreviations: BMI, body mass index; SMI, skeletal muscle mass index; MetS, metabolic syndrome; TG, triglycerides; CAD, coronary artery disease; HDL-C, high density lipoprotein cholesterol; BP, blood pressure; FPG, fasting plasma glucose
