# Supplementary material for: Metabolic Syndrome, Sarcopenia and Role of Sex and Age: Cross-Sectional Analysis of Kashiwa Cohort Study
Source: PLoS One. 2014 Nov 18;9(11):e112718. doi: 10.1371/journal.pone.0112718 (PMC4236117; doi:10.1371/journal.pone.0112718)
Supplement: Table S2 — Adjusted associations of metabolic syndrome components with individual sarcopenia components. (DOCX) [file pone.0112718.s002.docx]

Table S2. Adjusted associations of metabolic syndrome components with individual sarcopenia components^*†^

| Men | All |  | Old-old^‡^ |  | Young-old^‡^ |  |
| --- | --- | --- | --- | --- | --- | --- |
|  | beta (95% CI) | p | beta (95% CI) | p | beta (95% CI) | p |
| SMI |  |  |  |  |  |  |
| Abdominal obesity | -0.18 (-0.25, -0.11) | <0.001 | -0.11 (-0.24, 0.02) | 0.09 | -0.20 (-0.29, 0.12) | <0.001 |
| High TG | -0.08 (-0.14, -0.01) | 0.02 | -0.08 (-0.19, 0.04) | 0.19 | -0.08 (-0.15, -0.01) | 0.03 |
| Low HDL-C | -0.03 (-0.10, 0.03) | 0.30 | -0.08 (-0.19, 0.03) | 0.14 | -0.004 (-0.08, 0.07) | 0.91 |
| High FPG | -0.04 (-0.10, 0.01) | 0.09 | -0.04 (-0.14, 0.05) | 0.34 | -0.04 (-0.10, 0.02) | 0.16 |
| High BP | -0.01 (-0.10, 0.08) | 0.86 | -0.08 (-0.25, 0.08) | 0.32 | 0.03 (-0.08, 0.13) | 0.62 |
| Grip strength |  |  |  |  |  |  |
| Abdominal obesity | -0.91 (-1.77, -0.05) | 0.04 | ^†^ |  | -1.45 (-2.55, -0.35) | 0.01 |
| High TG | -0.57 (-1.32, 0.20) | 0.15 |  |  | -0.80 (-1.77, 0.18) | 0.11 |
| Low HDL-C | -1.28 (-2.05, -0.51) | 0.001 |  |  | -1.64 (-2.65, -0.64) | 0.001 |
| High FPG | -0.25 (-0.88, 0.38) | 0.43 |  |  | -0.006 (-0.81, 0.80) | 0.99 |
| High BP | 0.21 (-0.87, 1.29) | 0.71 |  |  | 0.17 (-1.19, 1.53) | 0.81 |
| Women | All |  | Old-old^‡^ |  | Young-old^‡^ |  |
|  | beta (95% CI) | P | beta (95% CI) | p | beta (95% CI) | p |
| SMI |  |  |  |  |  |  |
| Abdominal obesity | ^†^ |  | -0.09 (-0.20, 0.03) | 0.13 | ^†^ |  |
| High TG |  |  | -0.12 (-0.22, -0.02) | 0.02 |  |  |
| Low HDL-C |  |  | -0.01 (-0.09, -0.07) | 0.77 |  |  |
| High FPG |  |  | -0.06 (-0.14, 0.02) | 0.13 |  |  |
| High BP |  |  | -0.09 (-0.23, 0.06) | 0.24 |  |  |
| Grip strength |  |  |  |  |  |  |
| Abdominal obesity | -0.84 (-1.47, -0.22) | 0.009 | -0.39 (-1.37, 0.59) | 0.44 | ^†^ |  |
| High TG | -0.40 (-0.95, 0.15) | 0.15 | -0.48 (-1.34, 0.38) | 0.27 |  |  |
| Low HDL-C | 0.03 (-0.41, 0.47) | 0.89 | -0.04 (-0.76, 0.67) | 0.91 |  |  |
| High FPG | 0.02 (-0.43, 0.47) | 0.94 | -0.57 (-1.28, 0.15) | 0.12 |  |  |
| High BP | 0.30 (-0.29, 0.89) | 0.31 | -0.58 (-1.85, 0.70) | 0.37 |  |  |

^*^ Models are fully adjusted for age, height, weight, physical activity and food intake, with five metabolic syndrome components (abdominal obesity, high TG, low HDL-C, high FPG, and high BP) included simultaneously as independent variables.

^†^ Analysis was carried out only when the association between metabolic syndrome and any of the individual sarcopenia components was statistically significant.

^‡^ The young-old group refers to those aged 65 to 74, and the old-old group to those aged 75 or older.

Abbreviations: CI, confidence interval; TG, triglycerides; HDL-C, high density lipoprotein cholesterol; FPG, fasting plasma glucose; BP, blood pressure; SMI, skeletal muscle mass index
